# The worldwide impact of telemedicine during COVID-19: current evidence and recommendations for the future

**DOI:** 10.20517/ch.2021.03

**Published:** 2022-01-04

**Authors:** Stefano Omboni, Raj S. Padwal, Tourkiah Alessa, Béla Benczúr, Beverly B. Green, Ilona Hubbard, Kazuomi Kario, Nadia A. Khan, Alexandra Konradi, Alexander G. Logan, Yuan Lu, Maurice Mars, Richard J. McManus, Sarah Melville, Claas L. Neumann, Gianfranco Parati, Nicolas F. Renna, Philippe Ryvlin, Hugo Saner, Aletta E. Schutte, Jiguang Wang

**Affiliations:** 1 Clinical Research Unit, Italian Institute of Telemedicine, Solbiate Arno, Varese 21048, Italy; 2 Department of Cardiology, Sechenov First Moscow State Medical University, Moscow 119991, Russia; 3 Department of Medicine, University of Alberta, Edmonton, Alberta T6G 2R3, Canada; 4 Biomedical Technology Department, College of Applied Medical Science, King Saud University, Riyadh 11362, Saudi Arabia; 5 First Department of Internal Medicine (Cardiology-Nephrology), Balassa Janos County Hospital, Szekszard 7100, Hungary; 6 Kaiser Permanente Washington Health Research Institute, Seattle, Washington, WA 98101, USA; 7 Department of Clinical Neurosciences, Centre Hospitalier Universitaire Vaudois (CHUV), Lausanne 1011, Switzerland; 8 Department of Cardiology, Jichi Medical University School of Medicine, Tochigi 329-0498, Japan; 9 Department of Medicine, University of British Columbia, Vancouver, British Columbia V6T 1Z4, Canada; 10 Almazov National Medical Research Centre, Saint Petersburg 197341, Russia; 11 Department of Medicine, University of Toronto, Toronto, Ontario M5S 1A1, Canada; 12 Sinai Health System, Lunenfeld-Tanenbaum Research Institute, Toronto, Ontario M5G 1X5, Canada; 13 Center for Outcomes Research and Evaluation, Yale-New Haven Hospital, Section of Cardiovascular Medicine, Department of Internal Medicine, Yale School of Medicine, New Haven, Connecticut, CT 06510, USA; 14 Department of TeleHealth, School of Nursing and Public Health, College of Health Sciences, University of KwaZulu-Natal, Durban 4041, South Africa; 15 College of Nursing and Health Sciences, Flinders University, Adelaide, South Australia 5042, Australia; 16 Nuffield Department of Primary Care Health Sciences, University of Oxford, Oxford OX1 2JD, UK; 17 Division of Cardiology, Saint John Regional Hospital, Saint John, New Brunswick E2L 4L2, Canada; 18 Nephrologisches Zentrum Göttingen GbR, Göttingen 37075, Germany; 19 Department of Medicine and Surgery, University of Milano-Bicocca, Milano 20126, Italy; 20 Istituto Auxologico Italiano, IRCCS San Luca, Milano 20149, Italy; 21 Unit of Hypertension, Hospital Español de Mendoza, School of Medicine, National University of Cuyo, IMBECU-CONICET, Mendoza 5500, Argentina; 22 ARTORG Center for Biomedical Engineering Research and Institute for Social and Preventive Medicine, University of Bern, Bern 3012, Switzerland; 23 School of Population Health, University of New South Wales, The George Institute for Global Health, Sydney 2042, New South Wales, Australia; 24 Hypertension in Africa Research Team, South African Medical Research Council Unit for Hypertension and Cardiovascular Disease, North-West University, Potchefstroom 2520, South Africa; 25 The Shanghai Institute of Hypertension, Ruijin Hospital, Shanghai Jiao Tong University School of Medicine, Shanghai 200025, China

**Keywords:** COVID-19, telemedicine, telehealth, m-health, Africa, America, Asia, Australia, Europe

## Abstract

During the COVID-19 pandemic, telemedicine has emerged worldwide as an indispensable resource to improve the surveillance of patients, curb the spread of disease, facilitate timely identification and management of ill people, but, most importantly, guarantee the continuity of care of frail patients with multiple chronic diseases. Although during COVID-19 telemedicine has thrived, and its adoption has moved forward in many countries, important gaps still remain. Major issues to be addressed to enable large scale implementation of telemedicine include: (1) establishing adequate policies to legislate telemedicine, license healthcare operators, protect patients’ privacy, and implement reimbursement plans; (2) creating and disseminating practical guidelines for the routine clinical use of telemedicine in different contexts; (3) increasing in the level of integration of telemedicine with traditional healthcare services; (4) improving healthcare professionals’ and patients’ awareness of and willingness to use telemedicine; and (5) overcoming inequalities among countries and population subgroups due to technological, infrastructural, and economic barriers. If all these requirements are met in the near future, remote management of patients will become an indispensable resource for the healthcare systems worldwide and will ultimately improve the management of patients and the quality of care.

## Introduction

The public health emergency that resulted from COVID-19 increased public interest and demand for telemedicine worldwide and led to a rapid expansion of this care modality as an integral part of outpatient care delivery^[1]^. Concomitantly with a dramatic reduction of in-person consultations, a surge in the use of remote patient monitoring and televisits has been observed. At last, telemedicine has emerged worldwide as an indispensable resource to restrain the spread of the disease through improving the surveillance of patients, facilitating early identification, and allowing prompt management of infected people, as well as enabling continuity of care of vulnerable patients with multiple chronic diseases^[2,3]^.

However, to date, the use of telemedicine worldwide remains suboptimal due to a heterogeneity of solutions and differences in the quality of infrastructures and technologies, as well as the level of acceptance of patients and doctors across different countries. With the aim of providing an update of the status and use of different modalities of telemedicine during the COVID-19 pandemic (from early 2020 to the end of 2021), we reviewed the current literature and asked experts from all over the world to summarize their experiences.

The objective of the present paper is not to provide a comprehensive picture of the use of telemedicine worldwide but to report experts’ perspectives, mainly focusing on the management of patients with chronic conditions requiring strict surveillance during home isolation^[4,5]^.

## Main Applications of Telemedicine During Covid-19

The COVID-19 pandemic has favored the rapid adoption of digital solutions and advanced technology tools in healthcare. Among these solutions, telemedicine, tools based on artificial intelligence (AI), big data analytics, and mobile tracing apps for surveillance, were widely employed to diagnose, prevent, monitor, and treat individuals worldwide^[6,7]^.

**Figure 1:**
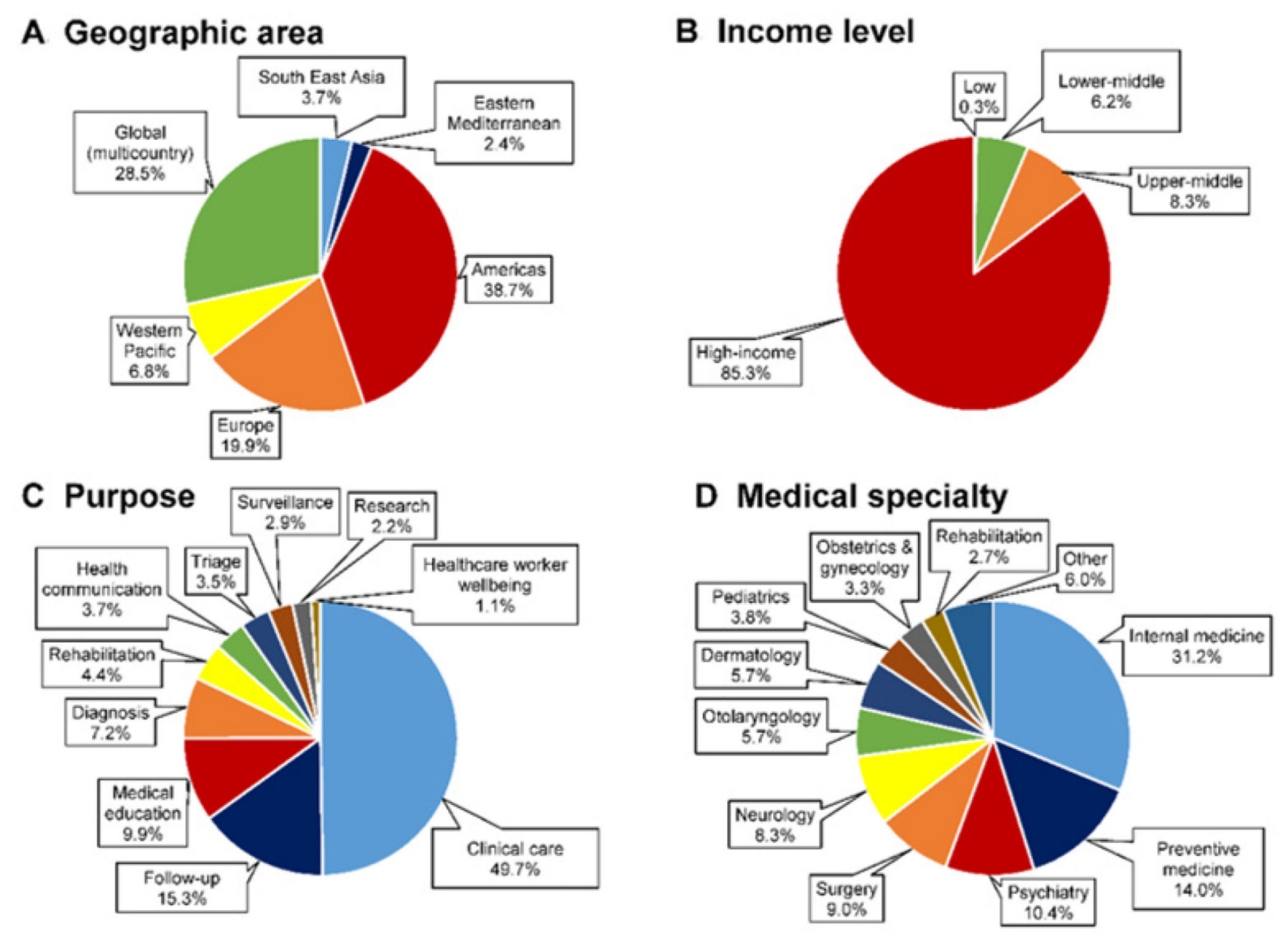
Geographic area (A), income level of the countries (B), purpose of telemedicine use (C), and medical specialty (D) of published articles included in a scoping review of the literature during the early COVID-19 pandemic. Redrawn with permission^[8]^.

A scoping review covering the early scientific literature in response to COVID-19 (January to June 2020) showed a wide use of telemedicine, principally in high-income countries (86.6% of the articles) [Figure 1]^[8]^. Of the 543 articles included in the review, the majority (92.3%) reported provision of telemedicine services for conditions not related to COVID-19, and only a few articles (7.7%) focused on the provision of services related to COVID-19. The majority of the articles focused on telemedicine use in internal medicine (mainly endocrinology, oncology, geriatrics, cardiology, and orthopedics), preventive medicine (for prevention of COVID-19, noncommunicable diseases and health education, in different settings, including primary care), psychiatry, surgery, neurology, otolaryngology, and dermatology. The most common purpose for using telemedicine was the provision of integrated clinical care (49.7%), including any combination of triage, diagnosis, treatment, follow-up, and rehabilitation services. Other purposes were follow-up care (15.3%), medical education (9.9%), diagnosis (7.2%), and rehabilitation (4.4%). The relevant role of telemedicine for the screening and managing of COVID-19 cases and the provision of continuity of care to chronic disease patients during the COVID-19 pandemic has been confirmed by other systematic reviews^[9–12]^.

During the COVID-19 pandemic, digital health and telemedicine solutions based on smartphone apps (so-called m-health) gained increasing popularity. Apps for digital contact tracings were used to collect and analyze data on people’s proximity, location, movement, and health status^[13]^. When used at scale, particularly when integrating AI-based technologies, these helped contain the spread of the virus and mitigate the effect of COVID-19 on population health^[13,14]^. Other m-Health applications were used for symptom checking, remote monitoring, and video consultation, and they were also valuable means for health information dissemination and awareness raising in the population. According to a systematic review conducted in the early phases of the COVID-19 pandemic, apps for contact tracings or symptom checking were prevalent in Asia, whereas the apps developed in Europe and North America remained focused on information^[15]^.

The specific experience of 15 countries or areas of the world in five continents are discussed in detail in the following sections.

## Experience In Different Countries

### Africa

#### North and mid Africa

The effect of COVID-19 in Africa has been different from most parts of the world. By July 2021, with fewer than 2% of the population fully vaccinated, the mortality rate in the Africa Region was 92 per million population. This is 5% of the mortality rate of the Region of the Americas, 1906 per million, and 7% of that of Europe, 1290 per million. Several possible explanations include a much younger population (the median age in sub-Saharan Africa is 18 years), few long-term care facilities for the elderly, insufficient testing, and prior exposure to local coronavirus strains^[16]^.

That Africa needs telemedicine is indisputable. The World Health Organization Health Report of 2006 noted, “…Africa has 24% of the burden (of disease), but only 3% of the health workers commanding less than 1% of world health expenditure”^[17]^. Unfortunately, the situation has changed little over the past 15 years as the population growth rate exceeds the production rate of health professionals. Thirty-one African countries have fewer than 20 doctors per 100,000 people^[18]^. Telemedicine has always been touted as a potential solution to some of Africa’s health problems, but there have been few successful and sustained telemedicine programs. Apart from the shortage of doctors and disease burden, the barriers to telemedicine are many, poverty, poor infrastructure, limited and expensive connectivity, and irregular power supply, to list a few^[19]^.

The COVID-19 pandemic has heightened interest in and awareness of telemedicine as in the rest of the world. As of September 2021, there were nearly 7500 papers on telemedicine and COVID-19 indexed in PubMed, 124 (1.6%) report activity in Africa, and, of these, only 28 (0.4%) report on actual telemedicine use during and in response to the pandemic.

Telemedicine activity is reported in 14 of the 54 countries, with most reports from Nigeria (seven papers). Eight of the papers are surveys of doctors in a particular specialty seeking to determine whether there had been uptake of telemedicine during the pandemic. These provide little detail of actual telemedicine services and the mode of telemedicine delivery. Overall, telemedicine activity has been reported in 16 clinical disciplines. The most common is mental health, reported in seven countries. Other examples include pediatrics, rheumatology, oncology, urology and obstetrics, and gynecology.

Given the generally poor information and technology and communications technology infrastructure in Africa, it is unsurprising that the telephone (10 papers), WhatsApp® (8 papers), and e-mail (2 papers) were the most common methods used. Videoconferencing using Skype, Zoom®, and WhatsApp® was reported in five papers.

Using e-mails and videoconferencing were unpopular because of technical limitations in Africa in general, and lack of insurance reimbursement for telemedicine and medico-legal and ethical concerns were noted as hindrances to telemedicine in many African countries. However, as elsewhere in the world, the COVID-19 pandemic will serve as a catalyst for telemedicine in Africa.

#### South Africa

Before COVID-19, telemedicine use was limited in South Africa, partly because of the lack of remuneration for telemedicine and, more importantly, the restrictive General Ethical Guidelines for Good Practice in Telemedicine in South Africa produced by the Health Professions Council of South Africa^[20,21]^. While their definition of telemedicine restricts it to communication between healthcare practitioners, doctor-to-patient consultation is allowed if there is an existing doctor-patient relationship.

South Africa has a two-tiered health system: state-funded public health serving roughly three-quarters of the population and the private sector, funded largely by individual contributions to medical aid/insurance schemes. Public sector doctors are salaried, while the private sector is fee for service based. Despite attempts to introduce telemedicine in the public sector over the last twenty years, telemedicine activity has been limited and largely confined to a few clinical and educational services provided by academic departments at medical schools. Radiology information systems, picture archiving systems, and laboratory information systems are used in both the private and public sectors. Informal telemedicine using instant messaging and videocalls between colleagues in both sectors is widespread but poorly documented.

COVID-19 has markedly increased telemedicine use in the private sector. Health professionals have resorted to telemedicine to overcome lockdown restrictions, triage patients, reduce unnecessary personal patient contact, and maintain an income^[22]^. This has been facilitated by temporary relaxation of the guidelines for the duration of the pandemic, allowing telemedicine to be practiced “*without an established practitioner-patient relationship*”^[23,24]^.

The private healthcare sector’s response was resilient and competitive. New services were rapidly developed, medical aids embraced telemedicine, and fledgling offerings were scaled up to cover COVID-19 triage, general consultation, rehabilitation, remote patient monitoring, and self-directed patient care. Teletriage reduced the burden on health facilities by saving 97% of users a face-to-face consultation. In a general practice service, 80% of problems were resolved by telemedicine consultation. Task shifting has occurred with nurses seeing patients in pharmacies and teleconsulting with doctors when necessary. Patients and practitioners have generally accepted these, with 97% of patients in one service willing to teleconsult again^[22]^. A novel service in the public sector reduced mortality in COVID-19 positive high-risk diabetic patients by about 20% by identifying the patients when diagnosed with COVID-19 and offering them preemptive hospital admission or daily telephonic follow-up for the first ten days after diagnosis^[25]^.

New issues have arisen. Payment for services varies from 55% to 100% of face-to-face fees and by discipline and telemedicine modality used - telephone or videoconference. Concerns about data ownership by medical aid schemes and interoperability issues of medical records are emerging in what has suddenly become a competitive market. Software developers and vendors are unhappy about practitioners’ use of free applications such as Zoom® or WhatsApp® for telemedicine.

What is the future? Many health professionals who were either ambivalent or opposed to telemedicine have become users. As elsewhere, will the enabling regulatory changes remain? If not, is there now enough demand and telemedicine experience, especially in the private sector, for practitioners and consumers to fight for change and the continuance of telemedicine?

### Americas

#### Canada

Virtual care delivery and remote patient monitoring (RPM) are the two most common telemedicine modalities currently offered in Canada^[26,27]^. Virtual care delivery typically consists of video (or telephone) visits between patients and providers, with multiple private companies offering virtual platforms that may include encrypted video connection, appointment scheduling, virtual waiting rooms, and document file sharing and storage. Mental health services, teledermatology, and primary care visits are the common indications for telemedicine use^[28]^. RPM is used primarily to monitor patients who have been discharged from acute care and/or admitted to virtual hospital programs, including chronic conditions such as hypertension, diabetes, chronic obstructive pulmonary disease, and heart failure^[27,28]^. This approach can be cost-effective if focused on high-risk patients^[29]^.

In the 1970s, Canada was a global leader in telemedicine^[30]^; however, in recent decades, prior to the coronavirus (COVID-19) pandemic, uptake of telemedicine has been extremely low. For example, in 2014, 411,778 telehealth clinical sessions were delivered, representing only 0.15% of the $270 million billable services provided nationally^[26,27]^. Conversely, virtual care visits have surged during the pandemic as Canadians have a high prevalence of connectivity. In 2020, 92% of Canadians had broadband access to the Internet, 69% used the Internet to search for health information, 84% used smartphones on a regular basis, and 14% of the population used Internet-connected wearable smart devices^[31]^. A study using population-based data analysis from Ontario, the largest Canadian province (population 14.6 million), found that virtual care use increased from 1.6% of total ambulatory visits in the second quarter of 2019 to 70.6% in the second quarter of 2020^[32]^. In a survey of family physicians in the province of British Columbia, telephone visits for hypertension increased from 18% to 80.7% during the pandemic^[33]^. In a 2021 survey of 2071 practicing Canadian physicians, it was reported that 94% of physicians were using virtual care and the most common modalities used were telephone (93%), video (51%), and secure e-mail/messaging (36%)^[34]^. Only 5% of physicians reported use of RPM platforms and 96% planned to continue to use virtual care in the future.

By the end of the summer 2021, more than 80% of Canadians have received the first dose of vaccine and 50% the second one^[35]^. With governments easing restrictions on movement and gatherings, few expect the health system will fully abandon virtual care as comfort using technology has grown, particularly among older adults. Instead, a hybrid model is envisioned in which healthcare services will transition to a “*flexible*” approach using digital technology to bridge the communication gap between clinic visits and provide a continuum of care. For health professionals, this means greater reliance on digital tools such as electronic health records, telehealth, and e-pharmacy, while, for patients, it means easier access to personal health records, expanded use of home monitoring devices, greater use of electronic messaging, and greater reliance on self-management, which is strongly promoted as a way to improve health outcomes and quality of care^[36]^. Thus, utilization of virtual care will likely increase and become the standard practice for persons with long-term conditions.

A nationwide survey in May 2020 showed that about 38% of Canadians considered virtual care to be the ideal first point of contact for healthcare services and 57% of Canadians reported using a remote modality (e.g., phone, text, e-mail, video, or virtual service) to obtain physician advice, with 91% satisfaction rates^[37]^. Despite widespread broadband access, substantial regional disparities remain, highlighting the digital divide in Canada. Broadband speeds in rural areas are 10%-20% of those in urban areas^[38]^, often below the useful connectivity speeds required for video conferencing, and one third of elderly Indigenous persons reported never using the Internet in a month^[39]^.

To facilitate the use of telemedicine services, a Virtual Care Task Force was established by the Canadian Medical Association, the College of Family Physicians of Canada, and the Royal College of Physicians and Surgeons of Canada^[40]^. The identified challenges and barriers to telemedicine uptake are those listed in Table 1.

Among the recommendations made by the task force for the implementation of virtual care in Canada, implementation of a national standardized framework to ensure the delivery of quality health services in virtual care was emphasized^[40]^. Other key recommendations included those listed in Table 2.

Overall, virtual care services should ideally be funded by the public sector in order to mitigate healthcare inequities. In addition, more stringent federal regulatory standards are required to prevent clinically inaccurate health monitoring devices from entering the consumer market, given that most of the devices on the market are not clinically validated^[41]^. This is a serious patient safety issue that should also be addressed as part of the development of a national virtual care program. It is also important that only clinically validated medical devices and Health Information Portability and Accountability Act (HIPAA)/Personal Information Protection and Electronic Documents Act (PIPEDA) compliant platforms should be used in order to ensure safe, quality healthcare service delivery.

#### United States of America

The utilization of telemedicine has rapidly expanded in the United States (US) in response to the COVID-19 pandemic. Data from the Centers for Medicare and Medicaid Services showed an increase in weekly telehealth visits from 13,000 pre-COVID to 1.7 million visits in the week of April 2020^[42]^. Compared with data from 2019, telehealth visits in October 2020 increased by more than 3000%. This unprecedented expansion of telemedicine occurred across nearly all medical specialties and included a broad range of services, such as video and audio-only visits, care chat, secure e-mail, and telemonitoring transfer of remote vital data. The increase was mainly fueled by reimbursement and policy changes^[43]^. During the pandemic, Medicare - the federal insurance program for the elderly in the US - rapidly expanded reimbursement for telehealth services and increased uptake of these services nearly overnight^[44]^. Additionally, the Department of Health and Human Services for Services Offices for Civil Rights waived penalties for HIPAA violations who were using everyday communication technologies to provide telehealth services^[45]^.

The utilization of telemedicine has had positive impacts on facilitating access to non-life-threatening healthcare, reducing risk for transmission of SARS-CoV-2, conserving scarce medical supplies, allowing rapid deployment of large numbers of healthcare providers, and supporting continuity of care when local hospitals and healthcare centers were unable to meet demand. However, access to telehealth has not been equal in all segments of the population. COVID-19 has highlighted the digital divide as a critical determinant of widening disparities in healthcare by race and economic status^[46]^. For example, pre-COVID-19 hypertension was cared for primarily at face-to-face visits, with non-white race, lack of health insurance, and access to care the major risk factors for poor control^[47]^. COVID-19 decreases in face-to-face visits have increased the importance of capturing blood pressure (BP) measurements remotely. In the second quarter of 2020, after COVID-19 stay at home orders were enacted in many states, ambulatory care BP assessments decreased by more than 50% compared to the second quarter of 2019^[48]^. Furthermore, unequal access to home BP monitors, smartphones, and broadband Internet could worsen BP measurement and control disparities^[49]^. In a survey of federally qualified health centers (community clinics caring for underserved populations), telehealth uptake was lower in Southern states, rural health centers, and those with staffing challenges^[50]^. Larger well-resourced healthcare systems such as Kaiser Permanente accelerated the development and implementation of telemonitoring programs for chronic conditions including hypertension, diabetes, and heart failure. However, the reach (number of people enrolled) of these programs is still limited because of limited resources for implementing population management (such as pharmacist and nurse medication management programs) and patient reimbursement for the purchase of home BP monitors. Partnerships with the industry hold promise for extending BP telemonitoring reach. Some vendors offer cellular enabled monitors that can directly transfer BP, weight, and other vital data to a website or an electronic health record without a smartphone or broadband Internet, which would improve access to and usability of chronic condition telemonitoring^[51]^.

As the COVID-19 pandemic continues, maintaining the expansion of telehealth is critical to providing access to care, and the facilitators and barriers to its implementation need to be carefully considered. Lieneck*et al*.^[52]^ performed a systematic review of implementation of telehealth services in the US during the COVID-19 pandemic and found 24 US articles focusing on multi-level facilitators and barriers to adoption of telehealth in ambulatory care settings [Table 3]^[53]^. These telehealth facilitators and barriers are not unique to the US.

Future challenges include potential redaction of telehealth policies, particularly those that ensure pay equity similar to face-to-face visits and restrictions of the type communications that will be reimbursed. Systems for engaging patients in telemonitoring and chronic condition programs, still in their infancy, have been sped up by COVID-19, but progress could slow once the pandemic recedes^[54]^. Strategies including greater outreach, education, and infrastructure support are needed to ensure access to telehealth in marginalized, vulnerable, and underrepresented populations.

#### Latin America

The adoption of telemedicine in Latin America (LATAM) in recent years has been extremely slow. Insufficient communication infrastructure, biases, and financial resources have hampered its use. The regulatory framework was also out of date. As of 2019, while 65% of hospitals in Chile used telemedicine, less than 30% of hospitals in Argentina, Costa Rica, Mexico, Peru, and Colombia were doing so^[55]^. Only Colombia and Mexico followed the recommendations of the 2015 Global Survey on eHealth Reports on Teledermatology, Telepsychiatry, Teleradiology, and Telepathology, and reported programs in all four specialty lines^[55]^. In Chile, physician resistance was considered the second most important barrier to telemedicine adoption^[56,57]^. Notably, countries with a comparatively more individualistic culture, such as Argentina or Uruguay, may be more ready to adopt telemedicine initiatives than culturally collectivistic countries such as Guatemala^[55]^.

During the COVID-19 pandemic, LATAM’s healthcare and social security institutions have intensified and sped up the adoption of telemedicine solutions^[58]^. A recent study projects that the LATAM telemedicine market, valued at more than 1.5 billion USD in 2019, will grow at more than 20.5% annually between 2020 and 2026^[59]^. The positive results in telemedicine use have brought about diversification of the care model in compliance with the challenges set out by the different organizations. In Ecuador, teleconsultations grew from 320 consultations in April 2020 to 1159 in May 2020. In Peru, 96% of health networks are currently developing teleconsultation processes, and teleradiology services have been set up. In Mexico, telemedicine has become an everyday healthcare process: in 2020, compared to 2019, telemedicine helped save 12,339 journeys for in-person visits and 10 million pesos of healthcare costs. In Uruguay, the healthcare system, purely based on a face-to-face model, is now progressively introducing remote medical consultations through videoconferencing. In Brazil, during the pandemic, an increase of 76.8% in demand for telephone consultations compared to the same period in 2019 has been observed, with a minority of requests (28.8%) arising from problems related to COVID-19^[60]^.

#### Argentina

In Argentina, the COVID-19 pandemic was a shock to the system. After the onset of the pandemic, a package of laws to validate electronic prescriptions was passed, and TeleCOVID, a strategy funded by the Pan American Health Organization and World Health Organization, was launched. By September 2020, the Senate had approved a new bill that would regulate telemedicine in the future.

With the introduction of mobility restrictions, the use of telemedicine increased dramatically, particularly among certain age and health groups. This may have paved the way for a wider acceptance of telemedicine after COVID-19. The impact of the restrictions emerged the week of March 13. By March 15, 2020, the government had closed international borders and ordered schools to suspend face-to-face classes. Five days later, the government declared a total lockdown, resulting in a sharp drop in the mobility of all kinds. During the week of March 13, the total number of calls and the number of calls from first-time telemedicine users increased by 233% and 226%, respectively, and the number of calls that resulted in prescriptions increased by 342%. This increase persisted through the subsequent months of blockages and even beyond. Indeed, while telemedicine use decreased slightly after restrictions were relaxed, it remained significantly higher than during the pre-pandemic period, indicating a potentially permanent shift towards acceptance of innovative medical practice^[61,62]^.

It was not just the number of new users that made the difference. The characteristics of telemedicine users also changed. Patients who used telemedicine during 2019 had an average age of 30 years with no underlying health problems: the substantial increase in the use of telemedicine during the pandemic was driven by patients 65 and older and those with pre-existing conditions.

For telemedicine to become a reality in the region, many problems will need to be addressed, including a more robust legal and regulatory framework for patient privacy, insurance, and professional licensing. In addition, in Argentina, in September 2021, the CHARGE-app study was launched: an iconic study in Latin America, through which it will be possible to evaluate the telemedicine strategy versus classic care in the management of hypertension^[63]^.

### Asia

#### Western Asia

In western Asia (Arab countries), each country and city had its own issues during the COVID-19 pandemic, but they faced broadly the same problems when administering care for patients with chronic conditions. Restrictions surrounding COVID-19 have led most countries to offer alternative systems to enable patients to access care during the pandemic, including telemedicine.

Clinics in Saudi Arabia and the other Gulf countries had varying degrees of access to and experience with utilizing telemedicine infrastructure prior to the COVID-19 pandemic. As the pandemic developed, these clinics had to pivot to offering remote services only. Most clinics in the Gulf countries turned to telemedicine to enact this, via methods such as phone calls, e-mail, Zoom®, and Microsoft Teams®, as well as smartphone-based technologies such as WhatsApp® and specific health apps^[64]^. They used these platforms for virtual visits and consultations, medication refills, rescheduling appointments, reviewing the measurements of vital signs, and so on^[65–67]^. Prior to COVID-19, most patients of these clinics had not used telemedicine platforms, but, due to high levels of access to smartphones and other devices and the wide availability of Internet in Saudi Arabia, relatively simple telemedicine clinics could be implemented quickly and smoothly, without too much additional burden being placed on the health system^[65]^. Research reported high patient satisfaction following attendance of a virtual session, and 88% of these patients recommended continuing this activity as a virtual session every year^[64]^.

In other western Asia countries, some digital services had been present prior to the COVID-19 pandemic, mainly involving phone line call centers and messaging services. However, some of these countries experienced difficulties in pivoting to a fully remote model of care during this time^[66]^. Illiteracy presents a potential barrier to successful telemedicine implementation. Even educated patients may still lack the technological literacy to engage with the communications platforms and other technologies required for telemedicine. Its implementation has also been severely hampered in countries that have experienced socioeconomic disruption, such as Libya and Syria, where technologies required for telemedicine cannot be utilized reliably due to lack of infrastructure, including the Internet and electricity^[66]^. These issues are particularly common outside of urban population areas.

#### China

The community lockdown due to the COVID-19 pandemic in China encouraged the use of telemedicine for both the prevention and management of COVID-19 and the management of chronic diseases. Three types of telemedicine platforms providing online services were adopted during the epidemic in China: (1) Internet hospitals operated by public hospitals; (2) Internet hospitals operated by online healthcare enterprises; and (3) local government digital service platforms^[68]^. The services provided included: (1) consultations services for COVID-19; (2) consultation services for derived health problems, particularly psychological counseling; (3) telemedicine and medical imaging teleconsultation; (4) general practice medicine for common and chronic diseases; (5) screening solutions to identify highly suspected COVID-19 patients, often based on AI; and (6) medical assistant robots.

According to a report from one of the largest hospitals in western China, from February 1 to April 1, 2020, 10,557 online COVID-19 consultations were conducted for 6662 individuals, and 32,676 patients without COVID-19 completed virtual follow-ups^[69]^. Of the 10,557 COVID-19 consultations, 487 individuals (7.3%) were suspected cases. After comprehensive testing, four individuals were confirmed cases (0.82%, 4/487). Of the 32,676 non-COVID-19 consultations, 10,981 received e-prescriptions through an app. In this large hospital alone, the monthly average of patients receiving online follow-up from February 1 to April 1, 2020 increased by nearly fivefold, from 3400 to 16,338. The situation was very similar across the hospitals and for various diseases in the whole country of China.

The use of telemedicine services was also widespread among physicians. A survey conducted among 148 physicians from 57 hospitals in 16 provinces across China revealed that 94.6% of them adopted a telemedicine system during the pandemic; 34.1% of the physicians had never used a telemedicine system before; and only 9.3% used it with a frequency of at least once per week^[70]^. Overall, 91.5% and 88.4% of physicians were willing to use telemedicine during and after the pandemic, respectively. Physicians considered the inability to examine patients in person to be the biggest concern (78.3%) and the most important hurdle (58.0%) to implementing telemedicine.

Hypertension is the most common cardiovascular risk factor observed in COVID-19 hospitalized patients. The COVID-19 pandemic and consequent lockdown sped up establishing an intelligent Hypertension Excellence Center system, an endeavor to use a telemedicine platform to ensure continuity of care for hypertension cases diagnosed before the pandemic^[71]^. In regional medical centers, physicians working with the platform are connected with their patients by using an app that collects clinic, ambulatory, and home BP data and other health information. Physicians may do electronic counseling and even prescription through the system. Millions of patients have been on the platform and hopefully improve their hypertension management and cardiovascular health before and during the pandemic. In addition, with the established telemedicine platform, the May Measurement Month project in China continued over 2020 and 2021^[72]^. Individuals measured their BP with a fully automated BP measurement system. BP was measured in about 100,000 and 300,000 people in 2020 and 2021, respectively, at more than 200 measurement sites in more than 20 China provinces. This opportunistic mass screening program commenced in 2017 worldwide, and helped more than one million Chinese people know their “*numbers*”^[72]^.

#### Japan

In Japan, since April 2020, the number of outpatients with chronic diseases such as hypertension, diabetes, and cardiovascular disease decreased in clinics and hospitals. Among 100 patients who answered a telephone survey in a university hospital in Tokyo during the COVID-19 pandemic, 20% reported depressive symptoms, and 33% were hesitant to contact medical staff in the event of cardiovascular disease exacerbation^[73]^. The frequency of depressive symptoms was sustained even after a decline in the number of newly COVID-19-infected patients. Following the rise of the COVID-19 pandemic, the Ministry of Health, Labor, and Welfare (MHLW) recommended the use of telemedicine. In April 2020, the government temporarily allowed the adoption of telemedicine for the first consultation between a patient and a doctor and the online prescription counseling by a pharmacist. After the first decision to temporarily expand telemedicine, the Japanese government sped up its pre-pandemic discussions on telemedicine and finally declared in June 2021 that these measures would be maintained permanently. MHLW will develop detailed guidelines to ensure the safety and quality of the treatment, followed by formal discussions on the reimbursement rate reform. Since the first decision about telemedicine, doctors were permitted to use telemedicine. However, almost all the clinics and hospitals were not prepared for sophisticated telemedicine applications. The majority of doctors used the telephone for teleconsultation as a basic form of telemedicine.

According to a survey by the MHLW in January 2021, the number of medical institutions which introduced telemedicine after the authorization of the government increased from 10,812 (9.7% of all institutions in Japan) in April 2020 to 16,095 (14.5%) in June^[74]^. However, this number nearly plateaued for the rest of the year. Initial online consultations for the first visit were less than 10,000 nationwide, even in the peak month of May 2020, and have decreased since. Both the usage and the rate of expansion seem somewhat limited compared to other high-income countries, such as the US.

Telemedicine was also popular among Japanese patients. In a large Internet survey conducted in two different periods during the COVID-19 pandemic in Japan, of 24,526 participants aged 18-79 years (50.8% women), 2.0% (*n* = 497) reported using telemedicine in April 2020 and 4.7% (*n* = 1159) during August-September 2020^[75]^. During the pandemic the use of telemedicine was increased more in younger than older individuals, except for subjects in their 70s who showed higher frequencies of telemedicine use. Inequality in telemedicine use by educational attainment and urbanicity of residence widened during the COVID-19 pandemic. Between August and September 2020, individuals with a university degree were more likely to use telemedicine than those with a high school diploma or less (6.6% *vs*. 3.5%). Residents in urban areas exhibited higher rates of telemedicine utilization than those living in rural areas only during August-September 2020 (5.2% *vs*. 3.8%). Disparities in telemedicine use by income level were not observed in either period.

In Japan, a web-based information and communications technology (ICT)-based BP monitoring system [the Disaster CArdiovascular Prevention (DCAP) Network] was developed and introduced in an area that was catastrophically damaged (Minamisanriku town) to help control the survivors’ BP in 2011^[76]^. This DCAP system was developed to help care for people in the disaster area. However, it was also used after the acute phase and contributed to increasing BP management quality, resulting in decreased cardiovascular events for the next ten years. In Minamisanriku town, the DCAP system has been used continuously, even during the COVID-19 pandemic, to keep improve BP control in hypertensive patients. This BP monitoring system with cloud computing was used for affected people staying in temporary shelters. Self-measured BP was automatically sent to the cloud computing system. At this early stage, just after the disaster occurred, the DCAP network system was helpful to pick up the high-risk patients with disaster hypertension and hypotension due to infection or dehydration. When the hypertensive patients returned to their houses after the acute phase of the disaster, the DCAP home BP monitoring system helped evaluate the home BP control status in the living conditions changed after the disaster. This DCAP home BP-guided approach helped decrease the participants’ home BPs from around 150 mmHg or more to significantly lower BP levels^[77]^. This DCAP network has been continuously used for ten years until now, and home BP has been successfully controlled with modest seasonal BP variation (120-130 mmHg), even without a change in antihypertensive medication. This system will be expanded to the community level as the second step to introduce the BP monitoring system into different settings.

The ICT technology is highly developed in Japan, and the Japanese Society of Hypertension generated the new research area of “*digital hypertension*”, promoting telemedicine in the clinical practice^[78,79]^. Telemonitoring and telemedicine will increase the accuracy of the diagnosis of hypertension and improve the assessment of BP control status. This will improve healthcare management and treatment for hypertension^[80]^. However, significant challenges remain: they are related to the cost, the data integration, the redesigning of team-based care, and better user experience in Japan.

### Australia

The first patient with COVID-19 in Australia was detected on January 25, 2020. To reduce the risk of patient-to-patient and patient-to-clinician transmission of COVID-19, the government responded quickly by introducing several tranches of new and temporary telehealth items to the national Medical Benefits Schedule on March 13, 2020. Compared to most countries, the rate of infections in Australia has been minuscule due to the government’s early actions to close the country’s borders and impose strong lockdown within districts when cases are detected. Followed by highly effective track-and-trace record keeping, the impact of the first two waves was small. However, the third wave with the Delta variant entered the country by the end of June 2021 and forced a strategy overhaul seeing for the first-time daily infection rates exceeding 1000 in August 2021.

Despite low numbers of cases and deaths in Australia, the previouly limited uptake of telehealth in the country changed rapidly with quick adoption, resulting in years of challenges for health system reform being achieved in a matter of days^[81]^. Overall, the introduction of “*whole-of-population telehealth*”, which includes remote consulting via telephone and video, is regarded as a major policy success.

**Figure 2:**
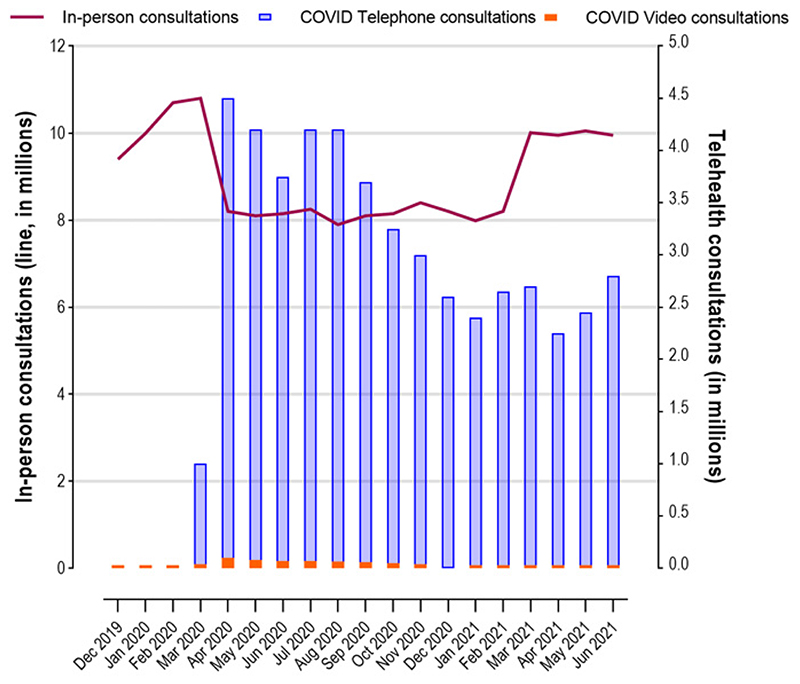
Trends in in-person, telephone, and video consultations with general practitioners during the pandemic in Australia. Reproduced with permission^[82]^.

Despite a policy emphasis on video telehealth as the preferred substitute for face-to-face care^[81]^, Australian healthcare providers demonstrated a clear inclination towards telephone rather than video consultations^[82]^. This has been especially true for general practitioners [Figure 2].

The reasons are not clear yet, but it may be pragmatic in nature, such as familiarity, need for rapid implementation, and availability of infrastructure. Other providers, such as psychiatrists and mental health consultations, were much more successful in incorporating video consults - often exceeding telephone consults^[82]^. Video was also a more common consultation method during the pre-COVID era, especially for psychiatrists and specialists^[82]^.

When it comes to quality of care and patient satisfaction, the telehealth environment seems less rosy. In an online survey of 683 surgeons^[83]^, 38% of respondents felt that the quality of care was equivalent to face-to-face consultations - with particularly the inability to perform a clinical examination a concern, and expressing that telehealth was an inappropriate means to break bad news^[83]^. In another survey, the clinician respondents felt that using telehealth services had been a “*forced adoption*” with many using telehealth for the first time^[84]^. Based on the responses, the authors concluded that, despite several positives, “*it could be that once the pandemic passes, previous policies and practises will re-assert themselves*”^[84]^. It is, however, often the acceptability of telehealth to patients that is the cornerstone on whether this mode of care has longevity. In a survey with outpatients that use only telephone consultations (88% aged 50 years or older), one in five indicated consistent dissatisfaction with telehealth. They often had lower literacy, qualifications, and access to the Internet^[85]^. Many other patients found teleconsultations convenient, but - similar to clinicians^[83]^ - there were concerns regarding absence of physical examinations. Patients expressed a dominant desire for a mixed-model clinic in the future^[85]^.

### Europe

#### Germany

As experienced worldwide, the COVID-19 pandemic increased the load on the German healthcare sector significantly. This situation accelerated the search for novel healthcare solutions, particularly for chronic illness care^[86]^. Developments focused on the introduction of telemedical solutions to the German healthcare sector on a broad scale. The Digitale-Versorgung-Gesetz (DVG), introduced in 2019 to facilitate innovation and digital transformation in healthcare, represented important legislation underpinning this process^[87]^. The DVG also contains the Digitale-Gesundheitsanwendungen-Verordnung, which regulates the reimbursement of digital healthcare applications through statutory health insurers^[88]^.

On June 16, 2020, the German government and the Robert-Koch-Institut released a COVID-19 warning smartphone app^[89]^. It recorded 28.6 million downloads in the first year. Since the initial release, additional features such as records of vaccination status and registration to events have been introduced. Furthermore, it was enabled to exchange data with similar applications across the EU. A significant change in practice during the COVID-19 pandemic was observed regarding video consultations. The joint federal committee (G-BA) updated the regulations governing video consultations to counter treatment gaps caused by a reduction in in-person consultations. Recent data show video consultations make up only 1% of cases. However, providers report an increase in demand by general practitioners^[90]^.

At the start of the pandemic, the state of North Rhine-Westphalia established a state-wide available intensive care hospital-to-hospital teleconsultation service (Virtuelles Krankenhaus NRW). Consultation requests are collected via a web portal, actioned via video conferencing, and documented in electronic case files (EFA)^[91]^.

As opposed to teleconsultations and teletherapy, telemonitoring solutions for chronically ill patients have not seen an increase in uptake in Germany to date. The opportunities for improved patient care provided by telemonitoring have yet to be recognized^[92]^. While there is excellent evidence for the efficacy of telemonitoring for some chronic illnesses, such as high BP^[93]^, Germany currently lacks (particularly monetary) incentive structures to join up existing initiatives and establish new ones.

Interest groups, such as the German Association for Digital Health Care (SVDGV) and the German Society for Telemedicine, and parts of medical societies work to improve the situation.

Telemedicine application in Germany falls short of full utilization of the technological possibilities. The acceleration of the adoption of telemonitoring and telediagnostics into the German healthcare system with appropriate recharge mechanisms for service providers is urgently required. Strengthening cross-cutting telemedical and digital health and care applications (DiGAs/DiPAs) is vital for the pandemic response and beyond. General practitioners and hospitals require low barriers to access telemonitoring infrastructure to further hybrid healthcare solutions and improve doctor-patient relations.

Recharge through statutory health insurers, education of all actors in the sector on evidenced digital healthcare applications, and the accelerated implementation of cooperation agreements between insurers and e-Health providers are the most important measures to improve the provision of telemedicine and telemonitoring across the sector. Here, the main focus needs to be on advancing already scientifically and economically proven telemedical applications.

#### Hungary

The COVID-19 pandemic has hit Hungary, as other European countries, hard during the second and mainly the third wave, claiming more than 30,000 lives, leading to one of the highest mortality rates in Europe considering the relatively small population of Hungary (313.32 deaths per 100,000 inhabitants). The subsequent restrictions, leading to limited personal access to medical care and general practitioners, made the optimal management and care of chronic diseases, such as hypertension, type 2 diabetes mellitus, chronic heart failure, or kidney diseases, more difficult. Despite the potential for telemedicine in this situation, the Hungarian telemedical providers and services were not developed or prepared enough to support and implement remote care, leading to initial missed opportunities. Nevertheless, some telemedical solutions were available in Hungary during COVID-19, notably the National e-Health Infrastructure (EESZT)^[94]^. This communication interface using cloud-based technologies connects public and private healthcare providers, pharmacies, and the population in the whole of Hungary. The EESZT was a significant milestone in the history of Hungary’s e-healthcare from the end of 2017. The system’s primary purpose is to interconnect the earlier fragmented healthcare data systems in all of Hungary and centralize all data in one system, taking into account foreign examples. Hence, the operating services of the infrastructure would allow access to the necessary information from the different treatment locations. Another important objective is to provide modern central services such as subsystems for issuing electronic receipts, electronic referrals and medical documents or e-imaging, or the e-profile, which facilitate the widespread adoption of modern healthcare.

Based on the EESZT, more subjects suffering from chronic conditions (e.g., chronic heart failure, hypertension, and type 2 diabetes mellitus) could access medical care and assess their condition during the pandemic. The main problem was sending their data [BP measurement, self-measured blood glucose values, and electrocardiograms (ECGs)] to their doctors. A basic solution was that the collected data of self-measured BP or blood sugar values were sent by e-mail from patients to their doctors, and the report or medical advice was sent back in the same way. The other options are mobile apps or smart devices capable of collecting and forwarding the measured BP readings. Unfortunately, an effective telemedical system is not yet established in Hungary, but the Hungarian Hypertension Registry employs special devices provided to family physicians and hypertension outpatient clinics. Office BP values are transmitted online to an evaluating center using the Medistance system of Omron^[95]^.

More and more patients with diabetes in Hungary use the so-called D-cont e-Diary system, which means smart self-measuring devices with the ability to upload blood sugar values to the Internet and an e-diary system. This can provide structured analyses to diabetologists (the last week, month, or longer duration statistics and different timepoint of measurements regarding fasting or nonfasting condition or carbohydrate intake), which results in better care of their patients.

Anticoagulated patients were also in a challenging situation during the pandemic, mainly those treated with vitamin K antagonists, because of the need for regular international normalized ratio checks and feedback on potential dose modification from their doctors. COVID-19 caused a switch in most patients to direct oral anticoagulant (DOAC) therapy, preferred because of the lack of need for regular blood checks. Modern telemedicine solutions could facilitate the e-prescriptions of DOACs, provide online educational materials, and solve perioperative management problems.

#### Italy

In Italy, the national health system struggled to sustain care for the wave of COVID-19 patients due to a shortage of personnel, devices, and intensive care unit beds. In April 2020, amid the national lockdown, the National Institute of Health (Istituto Superiore di Sanità) published interim recommendations on the use of telemedicine healthcare services during the COVID-19 health emergency^[96]^. The document supported the setup and implementation of telemedicine services, providing indications, identifying operational problems, and offering solutions supported by evidence that are easily implementable in practice. The recommendations were meant to be employed in different combinations to provide health services and psychological support and proactively monitor the health status of quarantined people, during isolation or after discharge from the hospital, or of people isolated at home due to the rules of social distancing but in need of continuity of care, even if they were not COVID-19 infected. Before the pandemic, the experience of telemedicine in Italy was anecdotal: services were traditionally scattered across several different applications, with poor interconnectivity and inconsistent local and regional reimbursement practices, and not covered by the national health system. General guidelines aimed to offer a guide for implementing telemedicine solutions were first released by the National Institute of Health in 2012^[97]^. However, these guidelines have never been practically applied. Now, following COVID-19, more practical recommendations on how to provide remote services, including televisits and telemonitoring, limited to patients with a chronic disease in stable conditions, have been issued^[98]^. To date, these indications still need to be put into practice.

During the first two months of lockdown (from March 9 to May 18, 2020), patients with acute conditions not related to COVID-19, or with chronic disease needing regular medical surveillance, could not easily access hospitals, general practices, or outpatient specialist clinics. This led to a dramatic reduction in hospitalization, an increase in the severity of the disease on admission, and an excess death rate in these patients^[99]^. The isolation at home also markedly increased the numbers of patients accessing health content on the Internet, as they were seeking qualified and certified telehealth services to help manage their condition. A significant increment in the use of home telemedicine solutions, particularly those based on m-health, has been observed during the lockdown due to the halting of face-to-face visits. The national health authority set up in March 2020 special patient monitoring units called USCA (Unità Speciali di Continuità Assistenziale or, in English, Special Units of Remote Care) in the frequency of one unit for every 50,000 inhabitants. These units included one or more physicians who could be activated at the request of the family doctor to perform interventions at the home of patients affected by COVID-19 but not requiring hospitalization^[100]^. This represented a rudimentary first attempt offered by the national health system to remotely manage, at scale, patients isolated at home. Concomitantly, local hospitals implemented or reinforced telemedicine services offered to Italian patients with different chronic conditions (including cardiovascular disease, diabetes, oncological disease, skin disease, neurological or psychiatric disease, and immunological disease)^[101–108]^. This “*health maintenance strategy*” provided individualized treatment and helped maintain the patient’s health status as close as possible to the ideal. Unfortunately, notwithstanding the recommendations of the National Institute of Health, the use of telemedicine by patients at home was hampered by the paucity of available services and the limited ICT infrastructure of the country.

**Figure 3:**
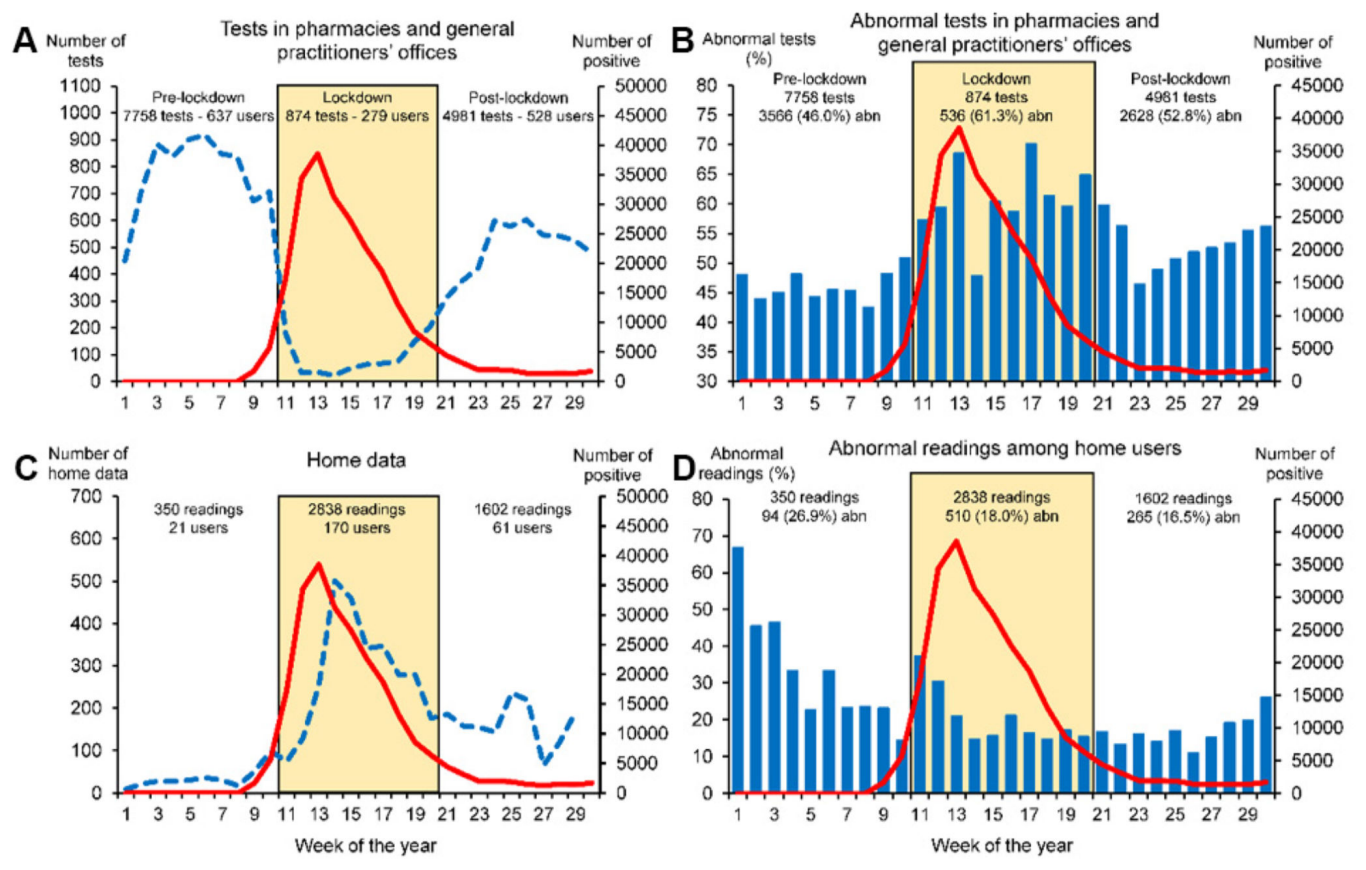
Weekly number of tests in pharmacies and general practitioners’ offices (A) and home data (C) collected before, during, and after the lockdown in Italy, and weekly frequency (%) of abnormal tests collected in the same settings and periods (B and D, respectively). Redrawn with permission^[109]^.

According to a recent publication, the number of home users of telemonitoring services and the number of data exchanged between patients and doctors increased during the pandemic [Figure 3C]^[109]^. The proportion of abnormal values (particularly for SpO_2_ and BP) was significantly lower during the lockdown [Figure 3D]. However, in the same period, access to existing telemedicine and telecounseling services provided to patients daily through community pharmacies and general practices showed a dramatic drop (ranging between 49% and 85%) because of the reduced access of patients to these premises due to fear of contagion^[109–111]^ [Figure 3A]. In the pharmacy setting, the overall proportion of abnormal tests was larger during the outbreak [Figure 3B]: in particular, an increase in the prevalence of abnormal ECGs due to myocardial ischemia was observed^[109]^.

#### Russia

The COVID-19 pandemic was a stimulus for telemedicine development in Russia as well as in other countries. Changes involved two spheres: (1) direct use of teleconsultations and distant monitoring for the treatment and monitoring of infected and contact subjects; and (2) use of telehealth for chronic diseases management (hypertension, heart failure, diabetes, *etc*.).

Telemedicine is a part of an extensive national program for healthcare informatization in the Russian Federation, which was highly accelerated by the current situation with COVID-19. The overall system has a part called “*Vertically Integrated Medical Information System*”, which is aimed to integrate all data from all regions in one block and analytics^[112]^. Currently, oncology and cardiology are two blocks that have already been introduced. The national registry of COVID-19 patients was quickly developed in April 2020, and now it is regularly updated by all regions: some of the data are downloaded directly from electronic medical records.

Limited to Moscow, and since April 2020, about 25% of outpatients with COVID-19 were managed by telemedicine services. A special telehealth center was organized for online consultations. Moreover, all chest computed tomography scans were collected in one large repository and analyzed by remote service. These innovations helped significantly reduce the load on outpatient clinics and improved the process of triage and organization of patients’ flow, including urgent hospitalization and ambulance visits.

In St. Petersburg, a specialized telemedicine system with the blocks of “*doctor to doctor*” and “*doctor to patient*” services was organized in 2020 for COVID-19. It included 18 specialties and more than 100 experts for specialized consultations, including intensive care in the critically ill. Two hundred fifty medical organizations are connected in the system, and the online interaction with patients and between hospitals is being actively developed.

BP telemonitoring with remote counseling intervention was organized in several regions of the Russian Federation. For example, in St. Petersburg, a simple free website and a mobile application were developed for patient-physician communication and storage and exchange of medical information. The design of the application and the telemonitoring process were previously described^[113]^. During the pandemic period, the usage of the system increased fivefold compared to 2019. BP telemonitoring solutions relying on different mobile applications and remote technologies have been implemented in 44 regions of the Russian Federation.

Each region introduced separate pilot projects, some of which were very successful. In Penza, e-Health services are currently used by 18% of the population. Online patients’ diaries were set up with about 8000 active users. Telehealth services are provided by state clinics and private organizations^[114]^, while more than half of activates are organized by non-medical providers such as Sberbank, Rostelecom, and others^[115,116]^.

#### Switzerland

Switzerland offers an excellent telemedical ecosystem, insomuch as several large commercial telemedical companies are headquartered in Switzerland^[117]^. With 24 h per day availability across the year, these services were already popular and widespread before the COVID-19 pandemic, with 2.5 million patient contacts per year^[118]^. Many major health insurance providers offer the “*Telmed model*”, requiring the insured to have a telehealth consultation before visiting a medical practitioner’s office^[119]^. This has led to almost half of all emergency hospital walk-ins to consult via a telehealth service before leaving their home^[120]^. As could have plausibly been expected, the number of Swiss health insurance providers offering a telemedicine consultation system for their customers rapidly increased during the first COVID-19 pandemic lockdown^[121]^. However, the rise in telemedicine use during the first lockdown did not involve individuals changing to telemedical services but rather using technology solutions to remain in contact with their primary care physicians^[122]^. Compared to commercial providers, the uptake of telemedicine by the wider Swiss medical community (e.g., general practitioners) was stagnating before the COVID-19. There were still several challenges to digitalization: (1) the lack of interoperability with their own systems; (2) security and liability concerns; (3) the inadequate representation of digital services in reimbursement tariffs; and (4) concerns about too much or too little work^[117]^. Unfortunately, these challenges do not seem to have markedly changed during the COVID-19 pandemic.

Nonetheless, the Swiss Association of Medical Doctors (FMH) has most recently been actively supporting the implementation of remote care services by assessing partnerships with telehealth manufacturers and by releasing a fact sheet proposing guidelines to the safe use of communication technologies and advice on billing for virtual consultations^[117,123]^.

During the first lockdown period, hospital care faced unprecedented challenges, such as reorganizing the hospital to treat patients with COVID-19. Consequently, there was a marked decrease in emergency hospitalization, such as myocardial infarction and stroke, in most areas of Switzerland^[124]^. Thus, as hospitals became overwhelmed by the global medical crisis, novel digital communication solutions circulated throughout Switzerland. For instance, the University Hospital in Geneva was in an ideal position to rapidly develop an application, HUG@home, by leveraging prior advancement of a pre-existing pilot version of a platform for telemedicine. HUG@home is particularly useful for vulnerable patients, such as those suffering from chronic conditions, and for communication between different healthcare professionals^[125]^.

Furthermore, progress has been made in cardiac rehabilitation, where telerehabilitation has become a standard procedure in larger cardiovascular treatment centers. The University Hospital in Bern now provides connected devices, such as BP and heart rhythm monitors, for remote diagnosis of cardiac insufficiencies^[126]^. Telemedical consultations are also increasingly being used by neurologists. For instance, in specialized epilepsy centers, teleconsultation, epileptic seizure monitoring with or without wearables, and Internet-based therapy programs, are commonly established either for everyday clinical practice or in the context of scientific studies^[127]^. Notably, the management of COVID-19 found direct support from the domain of telemedicine, with the country-wide downloads of the “SwissCovid App” used for contact tracing, which has strengthened the population’s trust in the security of healthcare data^[117,126]^. Other new technologies, such as the COVID-Guide, use artificial intelligence-driven chatbots for rapid self-assessment of symptoms and a personalized follow-up^[128]^.

#### United Kingdom

The COVID-19 pandemic had a major impact on the provision of care in the United Kingdom (UK), leading to significant reductions in services that have yet to recover: as of May 2021, estimates from the National Health Service (NHS) show that significant numbers were waiting for investigations or treatment in cardiology or cardiothoracic surgery in England - around 52,000 had been waiting longer than 18 weeks, and more than 4300 had been waiting for at least a year - up from just 28 before the pandemic started (at the end of February 2020)^[129]^. This reflects major problems for hospitals in undertaking their regular clinical activities alongside caring for those with COVID-19 or the consequences thereof. Despite these long waits, there have also been significant changes in activity in terms of admissions, presumably due to changes in behavior of both patients and clinicians: again, considering cardiovascular disease, hospital admissions for acute coronary syndrome declined from a 2019 baseline rate of 3017 admissions/week to 1813 per week by the end of March 2020, a reduction of 40% (95% confidence interval: 37%-43%)^[130]^. By May 2020, there had been a partial bounce back with 2522 admissions/week, representing a 16% (95% confidence interval: 13%-20%) reduction from baseline. This nevertheless represents significant changes in activity.

**Figure 4:**
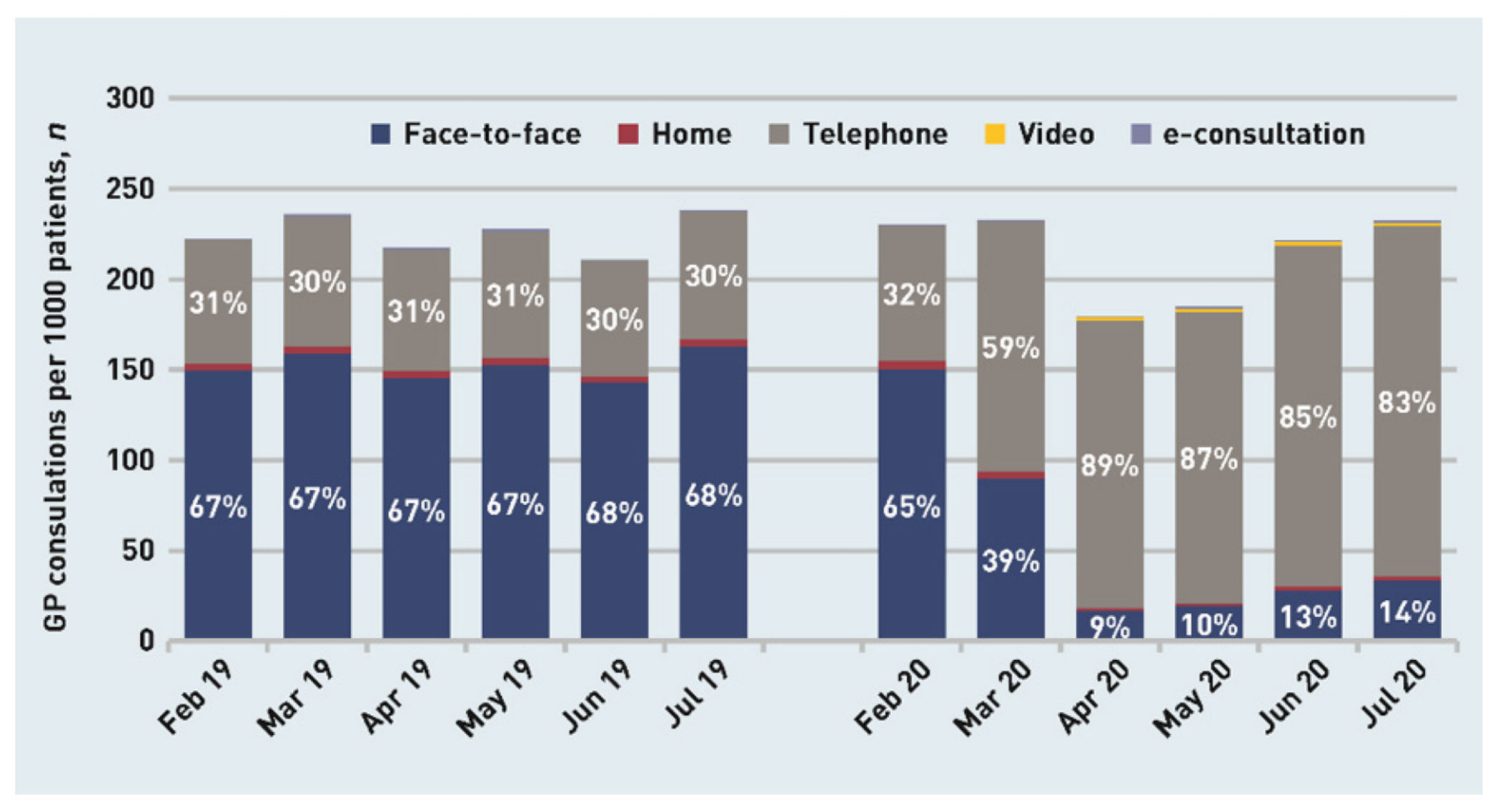
General practitioners (GP) consultations in the United Kingdom per 1000 registered patients, February to July 2019 and February to July 2020. Reproduced with permission^[131]^.

In primary care, there has been a rapid change to much higher levels of remote (largely telephone) consulting^[131]^ [Figure 4].

Despite relying on the trailing edge technology of a standard telephone or mobile, remote consulting jumped from 30% to 89% in the months immediately before and after the start of lockdown. In the UK, the use of short message service text messaging also increased by more than three times^[131]^. More complex technology was far more rarely used with very few video consultations undertaken, but patients often sent photos of, for example, rashes to allow remote management. Overall, consulting rates were also reduced in the pandemic by about 10% compared to pre-pandemic levels. Few data are available regarding case mix, but small increases were seen in the rates of consultation for mental health problems and by those who were shielding (the UK term for advice to self-isolate pre-vaccine availability for people in groups considered to be “*extremely vulnerable*” including those with transplants, significant cancer, and immunosuppresion)^[132]^.

To maximize cardiovascular prevention in the context of a large increase in remote consulting in UK, several initiatives have taken place. Self-monitoring of BP has been significantly enhanced by the BP@Home scheme: since October 2020, over 45,000 BP monitors have been delivered to people’s homes^[133]^. Early data from the trailblazer areas indicate that the level of home-based BP monitoring by patients, measured by the number of readings submitted, has increased three times compared to pre-pandemic levels. A further > 150,000 monitors have been ordered for distribution by the scheme (Personal Communication from NHS-X). Data published last year but collected pre-pandemic suggest that a further 40% of people with hypertension own monitors, which extrapolated to England as a whole would represent over three million people^[134]^.

In maternity care, NHS England procured 16,000 monitors, which were distributed to 127 hospital trusts nationally for use in pregnancy hypertension. This was accompanied by new guidance from the Royal College of Obstetrics and Gynaecology, providing advice on the use of self-monitoring in pregnancy for the first time in the UK. At least three providers offered apps to allow telemonitoring of results^[135–137]^. There are no current published data regarding these apps, although evaluations are in progress.

## Progress and Challenges in Telemedicine During Covid-19

The use of telemedicine during the recent pandemic has been rated as highly satisfactory by users, and, as a consequence, the majority of the patients and healthcare providers reported a willingness to continue using telemedicine after the pandemic^[138,139]^. In a global survey conducted in May 2021 involving 293 professionals across the healthcare delivery spectrum, 73% of respondents stated that telemedicine was the subsegment of digital health that experienced the largest growth during the pandemic^[140]^. In a previous survey conducted at the beginning of the pandemic, the percentage of positive responses was slightly lower (65%)^[140]^.

The situation relative to the adoption of telemedicine in the various continents or countries during COVID-19 as presented in the specific sections of this paper appears to be fragmented and heterogenous. The level of implementation, main barriers to uptake, primary services provided, and availability of insurance reimbursement plans and telehealth policies at a national level are summarized in Table 4. When insurance reimbursements or telehealth policies were available, these were limited to specific services and, in most cases, the pandemic period: in federal countries such as the US, the regulations may have varied from state to state.

As summarized in Table 5, during the COVID-19 outbreak, several steps forward in adopting telemedicine have been made. Although some pre-existing barriers to the pandemic were removed, important gaps still need to be filled in to favor large-scale implementation of telemedicine. Technological, infrastructural, educational, economic, and legal issues still undermine the long-term sustainability of telemedicine beyond the COVID-19 pandemic.

## Perspectives and Conclusions

During the COVID-19 pandemic, progress has been made in the adoption of telemedicine to screen for infected people, oversee affected subjects, and ensure continuity of care of chronically ill people. However, the use of telemedicine was not homogeneous across the various countries hit by the pandemic. This was due to differences in the awareness of the importance of telemedicine, variability in the quality of the infrastructures, level of informatics literacy of healthcare professionals and patients, and reimbursement schemes and plans. The experience collected during the COVID-19 pandemic should help strengthen available solutions and develop a more coordinated general strategy to favor the implementation of telemedicine at scale in the healthcare system. Achieving this goal will help prepare well for future pandemic waves and ultimately improve the management of COVID-19 and non-COVID-19 patients. In particular, second-line home-based telemedicine-based solutions serving as “*virtual hospitals*” may help reduce the burden on the first line, particularly emergency departments and intensive care units.

## Figures and Tables

**Table 1:** Challenges and barriers to telemedicine uptake identified by the Canadian Medical Association, the College of Family Physicians of Canada, and the Royal College of Physicians and Surgeons of Canada task force

Lack of physician reimbursement for virtual care visits Lack of virtual care services via the public health sector Lack of interoperability & compatibility of digital technology Governance of insured services and licensure restrictions

**Table 2:** Recommendations to ensure the delivery of quality health services in virtual care provided by the Canadian Medical Association, the College of Family Physicians of Canada, and the Royal College of Physicians and Surgeons of Canada task force

Establishing national standards for patient health information access Supporting federal regulatory authorities to simplify physician registration and licensure processes in order to enable virtual care services across provincial and territorial boundaries Developing revenue-neutral fee schedules for in-person and virtual care appointments Implementing education and training about virtual care technology for healthcare delivery in the undergraduate and postgraduate medical curriculum as well as providing continuing professional medical education on the topic for physicians and other healthcare providers Ensuring sufficient and equitable broadband access and speed in remote areas and other digital deserts and providing digital literacy training for the public

**Table 3:** Facilitators and barriers to the implementation of telemedicine in the United States^[53]^

Facilitators	Barriers
Patient engagement, including patient access to technology, education, family support, and interpreter services Organizational readiness, including workflow planning for remote visits, appointing, check-on, and standardized templates for documentation Regulatory and policy changes, including reimbursement parity as noted above	Patient limitations, including access to smartphones, computers, and broadband Internet, technology capability, and comfort with telehealth visits Clinical care issues including unavoidable deviations from clinical standards of care (such as patients rather than healthcare professionals taking vital signs, limited ability to do physical exams) Technology availability and training, weak information technology infrastructure, and inadequate reimbursement for non-clinical care costs

**Table 4:** Summary of the level and features of telemedicine uptake in the various continents and countries worldwide during COVID-19 pandemic

Continent/Country	Implementation level	Main barriers to implementation	Main services (in order of importance)	Insurance reimbursement	Specific telehealth policies at a national level
Africa	Low	Cost Infrastructure Connectivity Interoperability General and digital illiteracy	Telephone Social (chat) E-mail	No	No
Canada	Medium	Infrastructure Connectivity Interoperability Reimbursement Regulatory restrictions	Video Telephone RPM	Yes (partial)	No
United States of America	Medium/High	Cost Infrastructure Digital illiteracy	Video E-mail RPM	Yes (partial)	Yes (partial)
Latina America	Low	Infrastructure Privacy Regulations	Video Telephone	No	Yes (partial)
Western Asia	Low/Medium	Cost Infrastructure General and digital illiteracy	Telephone Video	No	No
China	High	• General and digital illiteracy	Telephone Video	No	No
Japan	Medium	Cost Interoperability	Telephone Video RPM	No	Yes (partial)
Australia	Medium	Infrastructure Digital illiteracy	Telephone Video	Yes (partial)	Yes (partial)
Germany	Medium/High	Infrastructure Education	Telephone Video	Yes (partial)	Yes
Hungary	Low	Infrastructure Digital illiteracy	Telephone E-mail	Yes (partial)	No
Italy	Low/Medium	Infrastructure Interconnectivity Reimbursement	Telephone Video RPM	No	Yes (partial)
Russia	Low	Infrastructure	Video RPM	No	No
Switzerland	Medium/High	Interoperability Security	Video RPM	Yes (partial)	No
United Kingdom	Medium	Infrastructure Digital illiteracy	Telephone Video	No	No

**Table 5:** Progress made in the implementation of telemedicine during the COVID-19 pandemic and gaps still to be filled in

Progress	Gaps
Several countries relaxed laws and regulations pertaining to the use of telemedicine (licensing of healthcare operators, privacy, reimbursement) Many countries have issued national guidelines and protocols guiding the implementation of telemedicine in the community Insurance companies and national health authorities started reimbursing expenses for patient care delivered via telemedicine Increased awareness of the usefulness of telemedicine among healthcare professionals and patients Increased adoption of telemedicine solutions (particularly televisit and telemonitoring) in the majority of countries worldwide More older patients with chronic diseases moved online compared to pre-COVID-19 Increased use of applications based on AI that may improve diagnostic accuracy and treatment, transforming healthcare management from passive to active or proactive Increased integration of telemedicine with traditional (in-person) healthcare services	Several countries are still affected by the lack of policy to legislate telemedicine Current guidelines are often generic and do not provide practical recommendations for the routine clinical use of telemedicine (target population, types of application, remuneration, *etc.*) There is no integration and standardization at an international level among protocols and guidelines Health reimbursement plans may not be available in some countries and may not be provided to all the different social strata of the population Funding frameworks for telemedicine in the context of public healthcare must be defined Illiteracy in low- and middle-income countries reduces the awareness about the importance of telemedicine Clinicians’ unwillingness to adopt telemedicine persists in some cases and needs to be overcome with adequate training and education The technological and infrastructural requirements and the high costs of telemedicine bear the risk of widening inequity among countries with different income levels and across various population subgroups Tailored solutions according to users’ features must be envisaged to scale up the use of telemedicine Most advanced solutions may not be affordable to all subjects and may be available only in high-income countries Quality of care in telemedicine is not always optimal compared to in-person care Heterogeneity of available solutions and technologies do not allow cross-platform interoperability and easy data exchange Reorganization of the healthcare network is necessary to switch toutpatient remote management

## Data Availability

Not applicable.
